# Preventing opioid overdose with peer-administered naloxone: findings from a rural state

**DOI:** 10.1186/s12954-019-0352-0

**Published:** 2020-01-09

**Authors:** Bridget L. Hanson, Rebecca R. Porter, Amanda L. Zöld, Heather Terhorst-Miller

**Affiliations:** 1 University of Alaska Anchorage, 3211 Providence Dr, Anchorage, AK 99508 USA; 2 University of Wisconsin-Stout, 712 South Broadway St, Menomonie, WI 54751 USA

**Keywords:** Opioid overdose, Opioid epidemic, Naloxone

## Abstract

**Background:**

In response to the opioid epidemic, naloxone distribution programs aim to prevent overdose death by making naloxone available and training people to use it. Peers of individuals at risk of opioid overdose are well-positioned to administer naloxone and prevent overdose death.

**Methods:**

We conducted key informant interviews with 18 individuals with past or current opioid and heroin drug use who had administered naloxone to a peer during an overdose emergency. Interviews explored individuals’ experiences with administration and their recommendations for program and policy improvement. Data were systematically coded and analyzed for themes.

**Results:**

Participants sought naloxone rescue kits because they perceived high risk of overdose. They described high satisfaction with training and felt prepared to administer naloxone during overdose incidents. Overwhelmingly, participants perceived naloxone to be effective and emphasized the need to make it widely available. Findings suggest that engagement in overdose prevention strategies other than naloxone differs by gender, with females more likely than males to use multiple different strategies. Participants described that overdose experiences do not have a lasting impact on drug use behaviors.

**Conclusions:**

Findings support the feasibility of naloxone distribution to peer opioid and heroin users and provide recommendations for policy improvement, including effective and well-advertised Good Samaritan laws and links to treatment for opioid use disorder.

Like Canada and Australia, the United States (US) is experiencing an opioid epidemic [1–3]. Simultaneously, many European Union, Asian, and African countries are seeing increased prescribing of, access to, and misuse of opioids, igniting concern of a possible pandemic [4–6]. In the US, the public health concerns associated with the opioid crisis are impacting every state. Although increases in opioid misuse began decades ago, overdose death rates have climbed markedly in the past few years, primarily due to fentanyl being increasingly mixed with heroin and other drugs [7]. In 2017, opioid overdose deaths in the US averaged more than 130 per day [3]. Additionally, beyond overdose, opioid misuse is related to increases in HIV and hepatitis C transmission, suggesting these related issues may be viewed as a syndemic [8].

Past research demonstrated that, in the event of an overdose, life-saving interventions can typically be administered within 1 to 3 h [9]. The administration of naloxone, an opioid overdose reversal medication, is the most effective way to prevent death during an opioid overdose. With the introduction and rise of fentanyl, timeframes for intervention are often shorter [10]. Therefore, best practice in harm reduction is to increase access to naloxone by supporting wide distribution [11].

For many years after naloxone was approved by the US Food and Drug Administration in 1971, emergency treatment of opioid overdose was restricted to use by emergency medical personnel such as hospital physicians and paramedics [12]. Over the past decade, as harm reduction efforts have focused on making naloxone more accessible and available in overdose situations, first responders like law enforcement have been equipped as well [13]. Law enforcement officers are frequently first to arrive on the scene of an emergency. When officers have and are prepared to use naloxone, administration can occur before other responders arrive, thus increasing the likelihood of effective overdose reversal.

However, challenges remain with giving first responders, including law enforcement, the responsibility of overdose reversal. One significant barrier is that people who use drugs are often hesitant to call emergency services when an overdose occurs due to fear of police involvement [14, 15]. First responders also report suffering emotional consequences due to increased numbers of overdose situations [16].

As an alternative to relying on first responders, distribution of naloxone to people who use drugs for administration by peers has been cited as a feasible way to prevent fatal opioid overdoses [17–19]. In the US, naloxone was first distributed to people who use drugs in the 1990s through underground programs in Chicago and San Francisco [20]. Though various initiatives were distributing the medication and access began increasing in 2010, it was not until July of 2017 that all 50 states and the District of Columbia had passed legislation to improve layperson access to naloxone. Strikingly, this level of layperson access in the US occurred roughly a decade after most developed countries [21]. Prior to lifting restrictions barring the general public from administering naloxone, several pilot studies were carried out to determine if peer users could be trained to administer naloxone in an overdose situation. In these pilot programs, needle exchanges primarily located in large cities were used as access points where simple kits were provided to people who used drugs. Early on, these kits contained an injectable form of naloxone prescribed to the individual that completed the training. Trainees were instructed to administer the naloxone, perform rescue breathing, and call 911 [22, 23]. These pilots demonstrated success in life-saving overdose reversals carried out by peers [18, 24]. One pilot, conducted by the Chicago Recovery Alliance, reported a 20% reduction in opioid deaths in Cook County during its first year [20].

Given the likelihood of peer users to be present during an overdose and the success of the pilot programs, providing naloxone to peers along with basic training on how to use it has been broadly implemented as a life-saving strategy. Analyses of the implementation of community-based opioid overdose prevention have demonstrated a positive association between distribution programs and decrease in opioid-related mortality [25]. For example, an eastern US study documented a 27–46% reduction in mortality from opioid overdoses with peer users responsible for 90% of overdose rescues [26] and another study examining decline in opioid deaths using a simulated 6-month period suggested that multiple distribution sites could lead to a 39.9% reduction in deaths [27].

Numerous studies have demonstrated that training the general public in naloxone administration increases knowledge on how to respond to overdose events, perceived confidence in responding, and willingness to carry out recommended procedures [12, 24, 28–31]. In addition to the clear benefit of increased knowledge and willingness to intervene, diffusion of information to peer users has also been documented. Injection drug users that participated in a prevention and distribution program reported sharing information provided by the training without prompting from the program, with some participants referring others to the program for training or requiring knowledge about how to administer naloxone as part of their “house rules” [32].

Historically, some concerns about potential implications of naloxone distribution have been presented by physicians and politicians alike. The primary concern is of a moral hazard—that people using drugs may seek to use greater amounts or stronger types, knowing that overdose reversal is possible [31, 33]. However, studies examining naloxone distribution have demonstrated mixed findings regarding these concerns. Some studies suggest that naloxone distribution may actually decrease use among users as much as 53% and increase treatment utilization as much as 25% [18, 34]. However, other qualitative inquiry has reported increased use among some due to the thought that there is a “safety net” [35].

Given the demonstrated effectiveness of naloxone distribution programs to peer laypersons as intervention for addressing opioid overdose, this study aimed to understand experiences administering naloxone and recommendations directly from naloxone end users in Alaska. As a rural northern state in the US, Alaska is uniquely positioned to contribute to this knowledge base for two reasons [36]. First, rural areas experience high rates of opioid misuse and overdose death [37]. Second, access to professional first responders and emergency medical services is more limited in rural areas than urban areas [38]. For these reasons, layperson access to emergency supplies and resources such as naloxone is critical for public safety and health.

## Method

### Program description

In February 2017, the State of Alaska Department of Health and Social Services (DHSS) began Project HOPE, an overdose education and naloxone distribution program. The project partners with community-based organizations to train laypeople and equip them with opioid overdose rescue kits. Brief training is delivered by staff or volunteers at the partner organizations and includes identifying signs of overdose and appropriately responding by calling 911, performing rescue breathing, and administering naloxone. The opioid overdose rescue kits include two 4-mg intranasal naloxone sprays (Narcan®), a face shield for rescue breathing, a pair of latex gloves, and an information card with instructions and additional resources.

Alaska had previously enacted legislation to increase access to naloxone and provide for certain immunities via a Good Samaritan law [39]. With the development of Project HOPE, Alaska’s DHSS chief medical officer also issued a statewide medical standing order for naloxone, marking the first time naloxone was available to the general public without an individual prescription.

### Participants

Key informant interviews were conducted with 18 laypeople who had obtained opioid overdose rescue kits from Project HOPE partner organizations and administered naloxone to a peer. See Table 1 for participant age, sex, race, and rurality characteristics. Rurality was categorized according to US Census Bureau definitions [40]; participants are described as rural if they participated at rural data collection sites or reported traveling from rural areas (i.e., more than 30 min) to urban data collection sites. Two participants reported that they did not have a permanent place of residence.

**Table 1:** Participant characteristics

Characteristic	Participants, *n* (%)
Age [years]	
21–30	2 (11.1)
31–40	6 (33.3)
41–50	2 (11.1)
60+	4 (22.2)
Unknown	4 (22.2)
Sex	
Male	11 (61.1)
Female	7 (38.9)
Race	
Alaska native	4 (22.2)
White/Caucasian	11 (61.1)
Mixed race	2 (11.1)
Unknown	1 (5.6)
Rurality	
Urban	12 (66.7)
Rural	4 (22.2)
Transient	2 (11.1)

Most participants (55.6%) had administered naloxone on more than one occasion. All but one participant reported current use of opioids, heroin, or methamphetamine; the remaining participant reported past use and was currently abstaining from drug use. All participants had administered naloxone to a peer within the past 12 months.

### Interviews

A semi-structured interview guide was developed by the authors. The guide was developed by prioritizing questions relevant for program feedback and was reviewed by program staff before use with participants. Questions focused on naloxone access, training quality, and naloxone use. Suggestions for improvements to naloxone distribution and overdose prevention were also solicited during the interviews.

### Procedure

Participants were recruited through word-of-mouth and advertisements at community organizations (e.g., syringe exchanges, homeless youth centers, health clinics) that distribute naloxone. Participants were recruited, and interviews were conducted in four different communities—two urban and two rural [40]. One of the urban locations drew participants from the immediate area as well as from surrounding rural areas within driving distance.

Confidential face-to-face interviews were conducted by two research team members in private spaces at the community organizations or accessible off-campus university locations. Informed consent was verbally presented, reviewed, and agreed upon immediately prior to each interview. Interviews lasted approximately 17 min (range = 8:05 to 27:15; *M* = 17:47), with length varying based on the amount of experience participants had using naloxone and the amount of time participants had available to be interviewed. Most participants completed individual interviews; two pairs preferred to be interviewed together. Safety measures were included to ensure participant well-being and confidentiality; information about local resources was shared with participants. Participants received compensation in the form of a gift card worth 40 or 50 USD (amount depended on locale and type of gift card) following participation. All procedures and the interview guide were approved by the University of Alaska Anchorage’s Institutional Review Board (IRB).

### Analysis

Interviews were audio recorded, transcribed verbatim, and then reviewed and edited for accuracy. The research team condensed data through first cycle coding [41] by creating a list of key domains corresponding to each interview question and developing a summary template. Two team members used the template to summarize the first two transcripts and ensure domains were identifiable in the data and captured consistently. The remaining transcripts were summarized and compiled into a domain matrix using Microsoft Excel for display and cross-checking by the second author [42]. Inconsistencies were discussed until consensus was achieved; revisions were made to clarify concepts. Domains were grouped into categories as data patterns identified through second cycle coding were added to the matrix [41]. Relationships and discrepancies within the data were highlighted within each category using constant comparison [43]. Emergent themes were identified and further explicated as key categories were reorganized; conclusions were tested and confirmed within the data [42].

## Findings

Findings are grouped into four distinct themes: naloxone availability, training, naloxone utilization, and emergency response.

### Naloxone availability

Participants described their experiences initially obtaining naloxone, overall accessibility of naloxone, and alternatives used during an overdose emergency when naloxone was not available.

#### Initial contact

A majority of participants learned naloxone was available through syringe exchange programs or through peers. Others heard through local media, health service providers, employers, or by observing naloxone being used during an overdose emergency.

Naloxone rescue kits were sought by many participants because they were active opioid users and recognized a personal vulnerability regarding overdose risk. Several had friends or family members who were opioid users and/or had witnessed an overdose emergency, as explained by one participant who stated, “people have a tendency to die in my apartment” (male, 68 years). The need to be prepared due to the risks of opioid use was emphasized by one individual who stated, “I always carry [a rescue kit] with me just because. I always have one in my backpack …” (female, age unknown). Others were concerned about the potential presence of fentanyl. One participant explained, “I’ve had other people OD on me and just, there’s a lot of problem with fentanyl around in dope and stuff” (male, 60 years). Overall, ensuring personal safety and safety for others by preventing an overdose death was a motivation for obtaining naloxone.

#### Accessibility

Participants described naloxone rescue kits as well-advertised and fairly easy to obtain, although some participants also noted that kits were occasionally out of stock. Many appreciated that organizations distributing naloxone had simple processes such as being able to, “...just go in there and ask for it” (male, 33 years) rather than, “...having to have some appointment and just some long process that makes it difficult to follow through” (female, age unknown). Most participants had positive interactions at community organizations when obtaining kits and found staff to be “knowledgeable,” “professional,” “friendly,” and “nonjudgmental.” The quality of experiences at the syringe exchange organizations made positive impressions. About the exchanges, one participant shared, “...if they sold [naloxone] at a store...I would still rather go to the needle exchange just because of how helpful they are and because they have experience” (male, 33 years).

Overall, travel time to obtain a naloxone rescue kit was minimal. Participants in urban settings described traveling up to 15 min by car or 30 min on foot while those individuals in more rural settings indicated travel times of up to 30 min by car. Those living in rural regions tended to obtain and maintain a larger supply of rescue kits (i.e., “several at a time,” “a whole bunch at a time”) compared to those in more urban regions. Many were able to receive multiple rescue kits at a given time to share with peers as secondary distribution was available to anyone who was trained and interested in supplying rescue kits to peers.

Participants identified few challenges in accessing naloxone. Barriers that were identified reflected both participants’ personal experiences and known peer experiences. The most commonly cited barrier was kits being out of stock or limited distribution hours. Other barriers, mentioned by a small number of participants, included fear of a confidentiality breach or a lack of desire to listen to training.

#### Naloxone alternatives

Generally, when people needed naloxone, they had a rescue kit available and did not require an alternative to reverse an opioid overdose. A small number of participants transported victims to the hospital or asked for naloxone “on the street” when they did not have it available.

### Training

Participants highlighted the value and appropriateness of the brief, verbal naloxone training provided by community organization staff when people received naloxone rescue kits. A majority of participants reported being satisfied with training and felt it to be “self-explanatory” or “enough to get by on.” Several participants appreciated the printed materials included in the kit, but a few did not find them necessary. One perspective included, “You don’t really look into a pamphlet to find out how to use it” (female, age unknown). Another explained, “I did glance at [the instructions]. It’s a no brainer – what you’re supposed to do with [naloxone]” (male, 43 years).

Participants offered suggestions related to training. One participant recommended that training emphasize physical positioning of the victim’s head, saying “It’s some things that are important...tilt the head back...a lot of people don’t realize that, but it does make a difference. It helps [naloxone] get through better” (male, 43 years). Another suggested training also included “something about not needing to go farther than the instructions, like putting someone in a bathtub of cold water” (male, 36 years). For those who lacked confidence administering naloxone during an overdose emergency, participants suggested opportunities for hands-on training or “refreshers” when obtaining a refill, including “just a quick conversation asking if they know how to use it” (female, 40 years).

While training is intended to be a requirement for obtaining a naloxone rescue kit to ensure a person knows how to use it during an overdose emergency, a small number of individuals refused naloxone training when obtaining a rescue kit due to lack of time or overconfidence. One participant who had declined to listen to training when obtaining a rescue kit reflected that training would have been helpful in order to use the kit properly during an overdose emergency. Another participant described using a friend’s kit and reading the printed instructions quickly during an overdose emergency in order to administer naloxone.

### Naloxone utilization

Participants described personal experiences using naloxone, perceived effectiveness of a rescue kit, and general overdose prevention behaviors.

#### Personal experiences

Participants were able to recognize opioid overdose signs and symptoms. Some described being the only person in a group who knew what to do during an overdose emergency. An experience of one individual who had administered naloxone was recounted as:

...my girlfriend at the time, one of her friends did a big, fat injection of heroin. He OD’d, and she called me freaking out. And everybody’s just standing around... “What do we do?” Nobody even bothered to call an ambulance. So I went there and gave him a shot up the nose of it and sat him up, got some cool rags to cool him down, made sure he was coherent. He snapped out of it pretty quick. (Male, age unknown.)

Prior to naloxone administration, many participants attempted to wake or stimulate victims in some way, although one participant shared that “I don’t waste a lot of time doing that, especially if the person isn’t breathing” (male, 60 years). Participants reported that, in addition to administering naloxone, rescue breathing was performed some of time, either by the participant or by others present during the overdose.

Participants most often used two 4-mg doses of naloxone nasal spray during an overdose emergency. Several recalled waiting up to 5 min after administering naloxone before giving a second dose. All victims awoke after receiving naloxone.

#### Perceived effectiveness

All participants perceived naloxone to be effective and described statements such as, “It’s instant” and “Nobody’s died.” One participant shared, “It’s a frightening situation but [naloxone] has always worked for me. It hasn’t failed me yet” (male, 60 years). Although effective to reverse an opioid overdose, several participants shared that naloxone did not impact substance use behaviors, other than a short-term reduction in use following an overdose event. One participant explained:

...I try to choose every day, but it’s not that easy. I’d like it to be. But [naloxone] does help. You’re just, like, in shock that that had to happen to you. And when you come back you are just so grateful...you are just, like, ‘If this [naloxone] wasn’t here, I would be dead.’ ...I want to live; everything in me does. But as far as just choosing not to use the drug, I wanted to use right after that. (Female, age unknown.)

A few participants thought people were less cautious about their drug use because of the availability of naloxone. According to one person, “People feel like they don’t have to be as careful when using when having [naloxone] around” (female, 43 years).

#### Additional overdose prevention strategies

Overdose prevention practices with groups varied across participants. For example, some individuals, especially those in rural communities, proactively designated a person responsible to administer naloxone if needed. Others informed peers about where naloxone was located in the room and reviewed how to use it. An approach taken by one was to occasionally ask peer users if anyone had [naloxone], “usually while we’re sitting around” (male, 43 years). Others did not usually discuss the topic except in cases when “...the heroin is strong” (female, 40 years). Another respondent indicated lack of planning and discussion about naloxone, saying, “I mean, you just try not to overdose, you know?” (male, 68 years).

Some participants made plans with fellow users prior to drug use to prevent overdoses including doing a test shot or suggesting that peers initially reduce the amount of drugs used in order to determine potency. One participant shared:

For me, myself, no matter what, I always just try a little bit and tell people around me to try a little bit first, because you can always do more. You cannot do less, you know? That’s what I try to tell people. (Female, 43 years.)

#### Gender differences in overdose prevention strategies

Interestingly, when describing overdose prevention strategies, all female participants discussed safety behaviors they had engaged in due to potency concerns such as taking a test shot, starting slow, and waiting for another person (frequently identified as a male significant other) to try the substance first. Female participants also described warning others about the potency of a supply. Fewer than half of the male participants mentioned potency considerations in regard to prevention, with responses focusing instead on naloxone as intervention by letting others know that naloxone is available, where to obtain it, and its location when using.

### Emergency response

Participants described their interactions with emergency services and first responders during overdose incidents. Overall, participants rarely called 911 during an overdose. Some stated they have never called 911; as explained by one participant, “Most of the time, people try to handle it on their own” (male, 40 years). Some participants described concerns about the potential impacts on their relationships with peers, as calling 911 “would be like being a snitch” (female, age unknown). A small number of those without a legal history or current legal involvement did call 911, particularly if feeling, “too nervous to handle the situation on my own” (female, 32 years). Most participants were distrustful of law enforcement and believed they would be treated “like criminals” or be charged with a crime for being part of the overdose situation. Participants were generally aware of the state’s Good Samaritan law but did not believe that it provided immunity.

### Recommendations for awareness and availability

When asked for final thoughts or recommendations, participant responses largely focused on issues of awareness and availability. Those who reported having administered naloxone only once attended to issues such as awareness and personal security. This differed from participants who have administered naloxone on more than one occasion, with all of those participants providing a recommendation addressing availability and universal prevention options like including naloxone in first-aid kits, passing naloxone out in hospitals, and having naloxone in every household.

## Discussion

The current study explores the experiences of laypeople obtaining naloxone rescue kits and administering naloxone to peers during an overdose emergency. Since these individuals are more likely than professional first responders to be present during an overdose and able to immediately administer naloxone, they can prevent overdose death. Overall, findings from the interviews highlight the feasibility of community-based overdose education and naloxone distribution, as participants reported high levels of accessibility and satisfaction as well as high perceived effectiveness of naloxone in reversing overdoses. These findings are consistent with other recent literature demonstrating the competence of peers in administering naloxone and reversing overdose [44] and also suggest that overdose education may be useful for overcoming opioid knowledge deficits experienced by rural individuals [45].

Participants indicated that the process of obtaining naloxone was relatively simple and their descriptions of the interactions with partner organizations suggest that these organizations were able to minimize barriers for access and treated individuals seeking naloxone with respect. Most partner organizations distributing naloxone are experienced agencies that have been providing other services, such as syringe exchange, to individuals who use drugs. This background likely well-positioned them to reach individuals at risk for overdose and in need of naloxone and enabled them to simply enhance their services to include naloxone, thereby providing a positive experience for their clients.

Rural participants in this study experienced more logistical challenges, such as transportation and travel time, in obtaining naloxone than urban participants. While services such as syringe exchanges are legal in Alaska and generally available in urban and surrounding areas, they are less accessible for rural individuals. The solution participants described was to obtain multiple kits at each visit to the partner organization and to share those kits with others. Rural communities experience a 45% higher rate of opioid-related overdose deaths than urban areas as well as a disparity in naloxone administration by emergency medical services [46, 47]. The current study suggests that providing individuals in rural communities with numerous kits for secondary distribution could be one way to help to overcome the shortcomings of relying on emergency services.

Participants indicated that experiencing overdose reversal with naloxone did not impact the use behaviors of their peers. While not specifically probed in the interview guide, participants also did not indicate that administering naloxone impacted their own use behaviors. Despite their experiences witnessing overdose emergencies and administering naloxone to peers, all but one of the participants reported current drug use. Therefore, harm reduction efforts may be bolstered by also enhancing a connection to treatment and recovery services, as suggested by previous research [18, 34].

Female participants described more caution than male participants in using drugs when risk associated with overdose was perceived to be higher than usual. Male participants, conversely, seemed to rely on naloxone rather than practicing other behaviors aimed to prevent overdose. This emergent difference in overdose prevention strategies by gender suggests that women may be more receptive to interventions aimed to reduce drug use or practice more conservative strategies and diffusion of information may be employed to extend those strategies to men. Increased willingness among females to engage in additional prevention strategies may be compounded by gender differences in the retention of knowledge about strategies, such as that demonstrated by a recent study where female trainees, compared to males, had higher levels of knowledge gained from naloxone training [48].

This gender difference in safety behaviors related to drug use mirrors gender differences related to treatment as men are more frequently referred to pharmacological treatments when seeking help compared to women who are more likely to receive a referral to psychotherapy [49]. This finding suggests that there may be a broader issue related to how interventions are presented based on gender. Further, it has been documented that women are more likely to seek treatment earlier than their male counterparts despite experiencing no significant difference in severity of use [49, 50]. A single underlying mechanism may drive women to both practice a wider variety of harm reduction strategies and seek treatment sooner than men. Given that multiple female participants in this study noted that they wait for their male partner to use first to test the strength of the drug, it is also possible that gender roles drive males to function in a protective capacity. As men experience higher rates of mortality from opioid overdose, further investigation is needed to better understand gender differences in overdose prevention behaviors and inform preventative strategies to reach men [51].

Participants’ experiences also provide information that is useful for policy improvement. Because the effects of naloxone are temporary, best practices for naloxone administration include seeking medical attention for continued care [11]. While calling 911 was a component of the training provided to participants, many of them reported experiencing barriers to contacting law enforcement. These barriers are well-documented and include fear of arrest, stigma, consequences related to housing, and custody [52]. While participants indicated familiarity with the fact that Alaska has a Good Samaritan law that is intended to provide immunity from prosecution, they did not trust it would be effective in protecting them. Revision of legislation and policy to enhance protections in overdose situations and promotion of those laws to bolster awareness could increase access to medical services and reduce mortality. However, policy improvements alone may not be sufficient [15]. Other strategies may include promoting community policing and EMS outreach in communities to improve the public’s perceptions regarding law enforcement and increase their likelihood of calling for help.

Finally, participants described the importance of naloxone availability and urged increased promotion, availability, and access of naloxone. Sustaining and enhancing naloxone availability is a critical harm reduction strategy. While recent nationwide overdose death rates indicate some decline and success in preventing overdose death, ongoing provision of naloxone is necessary for the millions experiencing opioid use disorder who are at risk for overdose and will not be able to experience recovery if they first die from overdose. Naloxone availability could be enhanced by combining naloxone with distribution of high-risk opioid prescriptions and increasing access via pharmacies in general by reducing the cost of naloxone sold for a cash price. As emphasized by participants in this study, it is critical for access to be simple rather than involve requirements that act as barriers (e.g., appointments, paperwork) as well as for the interaction to be free of stigma.

Findings must be interpreted in light of the study’s limitations. Despite efforts by the authors to recruit participants throughout the state, most participants came from the two largest cities in the state. Thus, the rural perspective may not be captured in its entirety. Additionally, participants were recruited through partner organizations and the majority of interviews were conducted at those locations. Individuals who obtain kits in other ways, such as through medical providers, may not be comfortable accessing community-based service organizations and may have different experiences obtaining and administering naloxone.

Future research might expand upon these findings by further exploring differences in experiences of peers, specifically differences based on gender and rurality. Such differences may have important implications for harm reduction and treatment strategies. For example, studies might explore ways to increase broad uptake of overdose prevention strategies described by females in this study (e.g., starting slow or testing small amounts). Other avenues of inquiry could examine diffusion of information from females to males. Additional research should also explore perspectives and experiences of groups other than peer users in obtaining and administering naloxone, such as first responders, law enforcement, and family members, including parents of young adult children.

## Conclusions

Naloxone availability is a crucial harm reduction strategy for the current opioid epidemic, and many states and communities are implementing and expanding naloxone distribution programs [53]. Peers of people at risk of overdose are most likely to be present during overdose emergencies and, especially in rural areas, may be the only person available to respond. Understanding these peers’ experiences administering naloxone and preventing overdose death informs strategies for harm reduction, ensuring programs can maximize effectiveness and reach. Experiences of these individuals can also be used to develop strategies to connect people who use drugs and are impacted by overdose, including themselves, to treatment and recovery services.

## Data Availability

All data collected and analyzed in this study are confidential and are not publicly available.
